# Conducting Polymer Mediated Electrical Stimulation Induces Multilineage Differentiation with Robust Neuronal Fate Determination of Human Induced Pluripotent Stem Cells

**DOI:** 10.3390/cells9030658

**Published:** 2020-03-09

**Authors:** Eva Tomaskovic-Crook, Qi Gu, Siti N Abdul Rahim, Gordon G Wallace, Jeremy M Crook

**Affiliations:** 1 ARC Centre of Excellence for Electromaterials Science, Intelligent Polymer Research Institute, AIIM Facility, University of Wollongong, 2500 Wollongong, Australia; evatc@uow.edu.au (E.T.-C.); qgu@ioz.ac.cn (Q.G.); snar683@uowmail.edu.au (S.N.A.R.); 2 Illawarra Health and Medical Research Institute, University of Wollongong, 2500 Wollongong, Australia; 3 State Key Laboratory of Membrane Biology, Institute of Zoology, Chinese Academy of Sciences, 100000 Beijing, China; 4 Department of Surgery, St Vincent’s Hospital, The University of Melbourne, 3065 Fitzroy, Australia

**Keywords:** induced pluripotent stem cells, conductive polymers, polypyrrole, electrical stimulation, differentiation, neuronal, ectodermal, mesodermal, endodermal

## Abstract

Electrical stimulation is increasingly being used to modulate human cell behaviour for biotechnological research and therapeutics. Electrically conductive polymers (CPs) such as polypyrrole (PPy) are amenable to in vitro and in vivo cell stimulation, being easy to synthesise with different counter ions (dopants) to augment biocompatibility and cell-effects. Extending our earlier work, which showed that CP-mediated electrical stimulation promotes human neural stem cell differentiation, here we report using electroactive PPy containing the anionic dopant dodecylbenzenesulfonate (DBS) to modulate the fate determination of human induced pluripotent stem cells (iPSCs). Remarkably, the stimulation without conventional chemical inducers resulted in the iPSCs differentiating to cells of the three germ lineages—endoderm, ectoderm, and mesoderm. The unstimulated iPSC controls remained undifferentiated. Phenotypic characterisation further showed a robust induction to neuronal fate with electrical stimulation, again without customary chemical inducers. Our findings add to the growing body of evidence supporting the use of electrical stimulation to augment stem cell differentiation, more specifically, pluripotent stem cell differentiation, and especially neuronal induction. Moreover, we have shown the versatility of electroactive PPy as a cell-compatible platform for advanced stem cell research and translation, including identifying novel mechanisms of fate regulation, tissue development, electroceuticals, and regenerative medicine.

## 1. Introduction

Living cells and tissues produce and respond to bioelectric potentials, which may vary depending on their developmental and pathophysiological state [1]. Suitably, the use of electrical stimulation in medicine has become well established, with electrical therapeutics available for various tissue types and clinical indications, including deep brain stimulation for treating Parkinson’s disease [2], transcranial direct current stimulation for the treatment of depression [3], and transcutaneous nerve stimulation for electroanalgesia [4].

Notwithstanding earlier recognition of the significance of bioelectricity and therapeutic stimulation, researchers have only recently begun to explore the potential of electric stimulus in vitro. Potential applications include augmented tissue engineering and tissue maturation, and in vitro modelling of in vivo electroceuticals [5]. Beyond laboratory-based research and development, resulting products—be they tissues or devices—may be clinically-amenable for tissue replacement and/or regeneration.

We and others have investigated electrical stimulation for modulating cell behaviour initially under conventional two-dimensional (2D)/planar culture and more recently for three-dimensional (3D) tissue engineering [5,6,7,8]. The 2D studies demonstrated stimulation biased native human neural stem cells (NSCs) and murine embryonic stem cells (ESCs) towards neuronal lineages [6,7]. More specifically, the stimulation of NSCs using electroactive PPy:DBS film or ESCs by electroporation of embryoid bodies (EBs) with or without conventional chemically-based inducers showed robust neuronal but less glial cell induction. Moreover, the differentiation of stimulated NSCs resulted in neurons with longer and more ramified neurites. The ESC differentiation was more rapid compared to traditional growth-factor based methods. Novel 3D electrical stimulation of human NSCs within a conductive bio-gel using printed polymer poly(3,4-ethylenedioxythiophene)-polystyrenesulfonate (PEDOT:PSS) pillar microelectrodes enabled the development and maturation of 3D human neural tissues with enhanced neuronal cell function and increased response to drug-induced disinhibition [5].

The advent of human pluripotent stem cells, initially through the discovery of human embryonic stem cells (ESCs) and then followed by iPSCs, afforded model systems to access early fate determination for basic and translational biology [9,10]. Somatic cell reprogramming to pluripotency has further advantages through the unrestricted production of patient-specific iPSCs [9]. In the field of neurology, iPSC-derived cells of the central and peripheral nervous systems, including neuronal subtype-specific cells and supporting glia, are enabling advances in understanding cellular mechanisms of inheritable diseases and delivering in vitro platforms for early phase drug discovery and regenerative medicine [11,12].

Conventional methods of directing the fate of ESCs and iPSCs into different lineages requires a variety of conditions involving changeable components such as chemical inducers with variable activities, not least because of batch variation. Consequently, there are inconsistencies that confound the reproducibility of the cell differentiation and the investigation of identifiable perturbations to understand and model diseases, develop and screen candidate drugs, and deliver cell-replacement therapy to support regenerative medicine. Here, we describe the novel use of facile and defined PPy-enabled electrical stimulation without conventional chemical induction to derive the three germ lineages—endoderm, ectoderm, and mesoderm from human iPSCs. Furthermore, the stimulation enabled a robust induction of neuroectoderm favouring neuronal fate determination. Our findings provide proof-of-concept for the use of electrical stimulation to standardise stem cell differentiation and add to the growing body of evidence for the in vitro and in vivo versatility of electroactive CPs such as PPy as stable and cytocompatible platforms for advanced stem cell research and translation.

## 2. Materials and Methods

### 2.1. Preparation of Polymer Films and Stimulation Modules

Polymer films were prepared as previously described [6]. Pyrrole (Py) monomer (Merck, Darmstadt, Germany) and dopant sodium dodecylbenzenesulfonate (DBS; Sigma-Aldrich, St Louis, MO, USA) solutions were prepared briefly with distilled and deionized water (dH_2_O; Sartorius, Goettingen, Germany). Gold-coated mylar (Solutia Performance Films, Canoga Park, CA, USA) was prepared for polymerisation by cleaning with isopropanol and dH_2_O, and then drying under a N_2_ stream. An aqueous monomer solution was prepared, consisting of 0.2 M Py and 0.05 M DBS, followed by degassing using N_2_ for 10 min prior to polymerization. PPy films were polymerized galvanostatically at a current density of 0.1 mA/cm^2^ for 10 min using an eDAQ EA161 potentiostat (Denistone East, NSW, Australia). The polymer growth was performed in a standard three-electrode electrochemical cell with the gold-coated mylar as the working electrode, a platinum mesh counter-electrode, and an Ag|AgCl reference electrode. After growth, the films were washed extensively with dH_2_O, gently dried with N_2_ gas, and stored under desiccated conditions until use.

**Figure 1:**
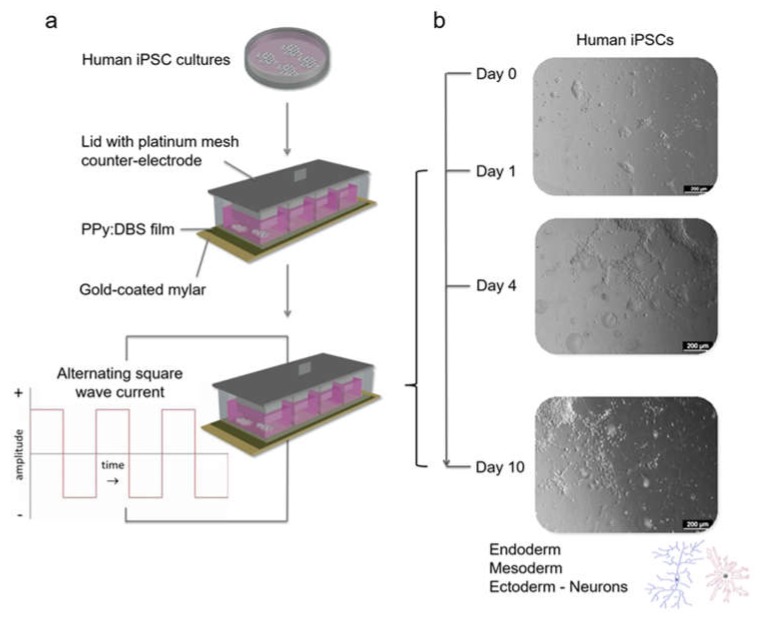
Schematic of the human iPSC stimulation setup and regimen using a PPy:DBS-based stimulation module: (**a**) Human iPSCs were harvested and transferred onto Matrigel^®^-coated PPy:DBS film within a stimulation module. Cells were stimulated in iPSC culture medium resulting in robust induction of neuroectoderm favouring neuronal fate determination. (**b**) iPSCs were initially maintained for 24 h (day 0–day 1) in culture medium before stimulating at a current density of ± 0.1 mA/cm^2^ for 8 h per day for 3 days (day 1–day 4), followed by ± 0.25 mA/cm^2^ for 8 h every 2 days for 6 days (day 4–day 10).

Stimulation modules were assembled by gluing clear bottomless polystyrene chambers onto the PPy:DBS films using silicon adhesive, followed by overnight curing at room temperature (RT) (Figure 1a). The lid of each module incorporated a platinum mesh electrode. The modules were sterilised with 70% ethanol (EtOH) for 20 min and air-dried in a biosafety cabinet, followed by storage under sterile conditions until use.

### 2.2. Cyclic Voltammetry

Cyclic voltammetry (CV) was performed using a CH Instruments 660D electrochemical workstation (Austin, TX, USA). The three-electrode cell consisted of a 3 cm^2^ PPy: DBS coated working electrode, a platinum mesh counter electrode, and an Ag|AgCl reference electrode. Scanning was performed in phosphate-buffered saline (PBS) at a rate of 0.1 V/s over a potential range of −0.7 to +0.7 V.

### 2.3. Human iPSC Culture

The human iPSCs (ATCC-BXS0116 and in-house derived JMC1i-SS9 lines; approved for use by the University of Wollongong Human Research Ethics Committee HE14/049) were cultured on a Corning^®^ Matrigel™ basement membrane matrix (Corning, Bedford, MA, USA) in mTeSR™1 (STEMCELL Technologies, Vancouver, BC, Canada) within a humidified 5% CO_2_ incubator at 37 °C. The medium was changed every 2 days. Cell passaging was performed at ~70% confluency by dissociating colonies with 0.02% ethylenediaminetetraacetic acid (EDTA; Sigma-Aldrich) at 37 °C for 2–3 min, rinsing twice with 1 mL prewarmed Dulbecco’s Modified Eagle Medium/Nutrient Mixture F-12 (DMEM/F-12; Gibco Life Technologies, Grand Island, NY, USA) and seeding dissociated cell aggregates at a 1:4–1:6 split ratio.

### 2.4. Conventional Human iPSC Differentiation

Conventional neural differentiation of the iPSCs was performed by replacing the mTeSR™1 culture medium with STEMdiff™ neural induction medium (NIM; STEMCELL Technologies) supplemented with a 10 μM Rhoassociated Coil Kinase inhibitor (ROCK inhibitor, Y-27632; STEMCELL Technologies). The cells were maintained for 3–4 passages with medium changes every 2 days. For longer-term maintenance of differentiated cells, the neural induction medium was replaced with STEMdiff™ neural progenitor medium (NPM; STEMCELL Technologies). 

### 2.5. Electrical Stimulation

PPy:DBS films within stimulation modules were coated with Matrigel^®^ at 4 °C overnight before use. iPSC colonies were mechanically dissected into small pieces that were transferred onto the films in either pre-warmed neural induction medium with a ROCK inhibitor (i.e., for conventional stimulation with chemical induction) or mTeSR™1 (i.e., for stimulation without chemical induction), followed by culture for 48 h or 24 h respectively, within a humidified 5% CO_2_ incubator at 37 °C. Stimulation was subsequently performed with the auxiliary platinum mesh electrode in contact with the culture medium. For conventional stimulation studies, stimulation was performed according to our previously published regimen [6], at a current density of ± 0.25 mA/cm^2^ for 8 h per day for 3 days, while for stimulation without chemical induction, stimulation was initially at a current density of ± 0.1 mA/cm^2^ for 8 h per day for 3 days, followed by ± 0.25 mA/cm^2^ for 8 h every 2 days for 6 days (Figure 1b). The cells were stimulated using a biphasic waveform of 100 µs pulses with 20 µs interphase open circuit potential and a 3.78 ms short circuit (250 Hz) using a Digital Stimulator (DS8000) and A365 Isolator units (World Precision Instruments, Sarasota, FL, USA) interfaced with an e-corder system (eDAQ). In response to the current pulse applied, the voltage waveform across the electrode area was recorded and total impedance (Zt) was calculated from maximum voltage (Vt) divided by the measured current output (i) (Zt = Vt/i). The access resistance and polarization impedance resulted from the initial voltage drop (Ra = Va/i) and the remaining voltage drop (Zp = Vp/i), respectively.

### 2.6. Immunocytochemistry and Analysis

The cell samples were fixed with 3.7% paraformaldehyde (PFA, Fluka; St Louis, MO, USA) in PBS for 10 or 20 min at RT. The samples were then blocked and permeabilized for 1 h at RT in 5% (v/v) donkey or goat serum in PBS containing 0.3% (v/v) Triton X-100 (Sigma-Aldrich). The samples were subsequently incubated with primary antibodies in a blocking buffer for octamer-binding transcription factor 4 (OCT4; mouse; STEMCELL Technologies), sex determining region Y-box 2 (SOX2; rabbit; Millipore, Temecula, CA, USA), stage-specific embryonic antigen 4 (SSEA4; mouse; STEMCELL Technologies), neuron-specific class III β-tubulin (Tuj1; mouse; Covance, Princeton, NJ, USA), and neuroectodermal progenitor cell markers Vimentin (chicken; Millipore, Temecula, CA, USA), Pax6 (rabbit; Sigma Aldrich), and nestin (mouse; STEMCELL Technologies), or Alexa Fluor^®^ conjugated antibodies for Tuj1 (mouse; BD Biosciences, San Jose, CA, USA), glial fibrillary acidic protein (GFAP; mouse; BD Biosciences) and nestin (mouse; BD Biosciences) at 4 °C overnight. The cells were then washed 3 times for 5 min with 0.1% Triton X-100 in PBS. For unconjugated primary antibody incubation, samples were incubated with Alexa Fluor tagged secondary antibodies (Life Technologies, Eugene, OR, USA) for 2 hr at RT. The cell nuclei were labelled with either 4’,6-diamidino-2-phenylindole (DAPI; Life Technologies) or Hoechst 33342 (Life Technologies) at RT for 10–15 min. The polystyrene chambers were carefully removed and ProLong^®^ Gold Antifade reagent (Life Technologies) was used to preserve fluorescence intensity. The samples were then coverslipped and imaged using a Leica TCS SP5 II confocal microscope (Leica Microsystems, Wetzlar, Germany). Images were collected and analysed using Leica Application Suite AF (LAS AF) software (Leica Microsystems) and Fiji (Image J; version 1.52p) [13]. The integrated density (IntDen; product of mean grey value and area) was measured for 3–5 biological replicates per marker and corrected for the number of cells per field of view.

### 2.7. Flow Cytometry

The iPSC colonies were dissociated using TrypLE™ Select (Gibco Life Technologies) for 5 min, washed in DMEM:F12 and centrifuged at 300 g for 5 min before removing the supernatant and fixing with 3.7% PFA in PBS on ice for 10 min. The cells were washed again, followed by further centrifugation at 300 g for 5 min. After blocking and permeabilizing in 5% goat serum plus 0.3% Triton-x-100 in PBS for 30 min on ice, the cells were incubated with primary antibodies diluted in 5% goat serum/PBS for 30 min on ice. Following a further 2–3 washes, the cells were incubated with species-specific secondary antibodies conjugated to Alexa Fluor (Life Technologies) in the dark for 30 min on ice. For conjugated antibodies, only one antibody incubation step was required. The wash protocol was repeated and the cells were resuspended in 2% FBS/PBS and analysed by a BD Accuri™ C6 flow cytometer (BD Biosciences, Ann Arbor, MI, USA). The antibodies used included: mouse anti-OCT4 (STEMCELL Technologies), mouse anti-SSEA4 (STEMCELL Technologies), mouse anti-TRA-1-60 (Millipore), and mouse anti-TRA-1-81 (Millipore). Isotype controls included anti-IgG2b, anti-IgG3, and anti-IgM. All isotype control antibodies were obtained from Life Technologies.

### 2.8. Real-Time Quantitative PCR (RT-qPCR)

For RNA isolation, the cell cultures were first treated with TrypLE™ Select (Life Technologies) and centrifuged at 190 *g* for 3 min. The supernatant was removed and the cell pellet was resuspended in TRIzol™ Reagent (Life Technologies; ~1 mL per 1 × 10^6^ cells) followed by the precipitation of RNA using isopropanol. RNA purity and quantity were assessed using a NanoDrop™ 2000c spectrophotometer (Thermo Scientific, Wilmington, DE, USA). cDNA was synthesized from the RNA templates using random primers. RT-qPCR was performed using a Gotaq 2-step RT-qPCR kit (Promega, Madison, WI, USA) and Bio-Rad CFX Real Time PCR instrument (Bio-Rad, Hercules, CA, USA). CFX software (Bio-Rad) was used to analyse data according to delta-delta Ct method [14]. Primer sequence information is provided in Table 1.

### 2.9. Statistical Analysis

Statistical analyses were performed in OriginPro 2015 (Version b9.2.272; Northampton, MA, USA) using one-way analysis of variance (ANOVA) with a Bonferroni multiple comparison post-hoc test. Homogeneity of variance tests (Brown–Forsythe Test) were performed to confirm that the statistical assumptions for ANOVA were satisfied. No statistical methods were used to predetermine sample sizes, and experiments were replicated to appropriately reduce confidence intervals and prevent errors in statistical testing. The collection and analysis of data was carried out unblinded to the conditions of experiments, with randomisation of study samples not performed. No exclusions were made. 

## 3. Results

### 3.1. Polymerisation, Electrochemical Activity and Stability of PPy:DBS Films 

**Figure 2:**
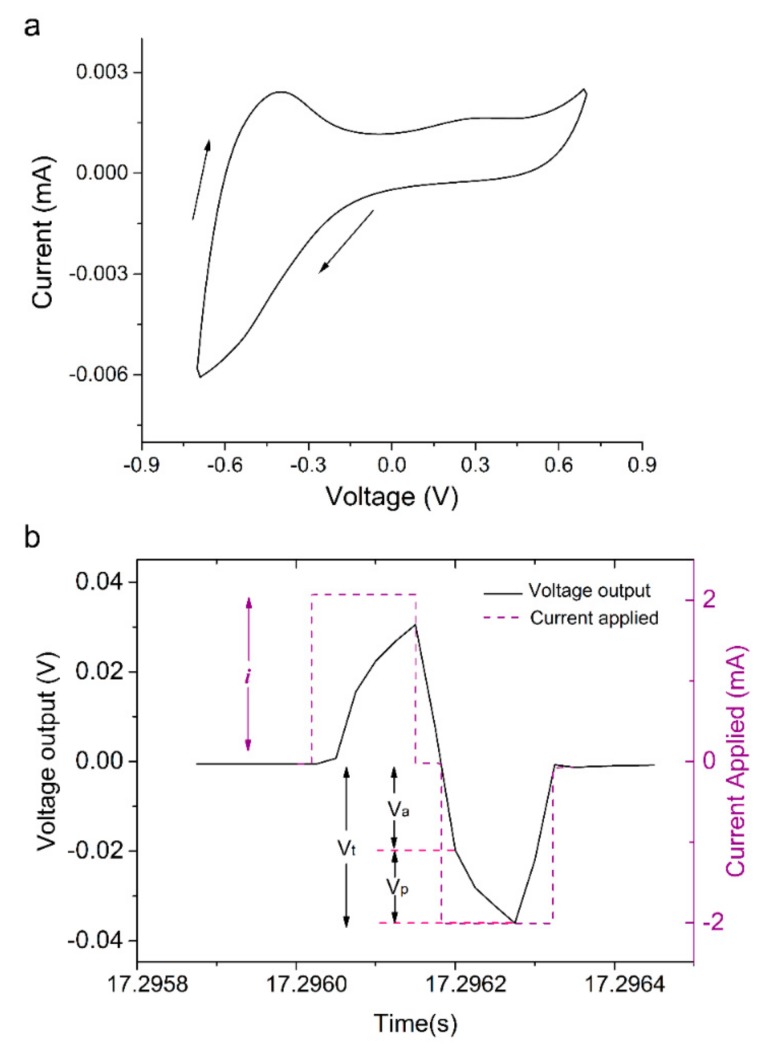
Electrochemical activity and stability of PPy:DBS film employed for iPSC stimulation: (**a**) CV voltammogram for PPy:DBS substrate in PBS electrolyte, with clear oxidation and reduction peaks indicating the presence of a redox active polymer. Arrows indicate the direction of potential scan (scan rate: 0.1 V/s, over a potential range of −0.7 to +0.7 V). (**b**) An example of the biphasic current (i) waveform applied, overlayed with the output voltage waveform used to calculate impedances, with Vt, Va and Vp denoting peak voltage output, initial voltage drop, and remaining voltage drop, respectively.

Applying the electropolymerisation conditions outlined above, uniform and adherent PPy:DBS films were deposited on gold-coated mylar substrates. CV demonstrated the electrochemical activity of PPy:DBS films with CV curves showing clear oxidation and reduction peaks, signifying a redox-active polymer (Figure 2a). The electrochemical stability of the films was confirmed by monitoring impedance during stimulation. Three impedance values were obtained from the voltage waveforms (total impedance: Zt = Vt/i, access resistance relating to changes in electrolyte: Ra = Va/I, and polarisation impedence relating to changes at the electrode surface: Zp = Vp/i) and showed negligable variation during the stimulation period (Figure 2b) [6,15]. 

### 3.2. Characterisation of iPSC Pluripotency and Differentiability 

**Figure 3:**
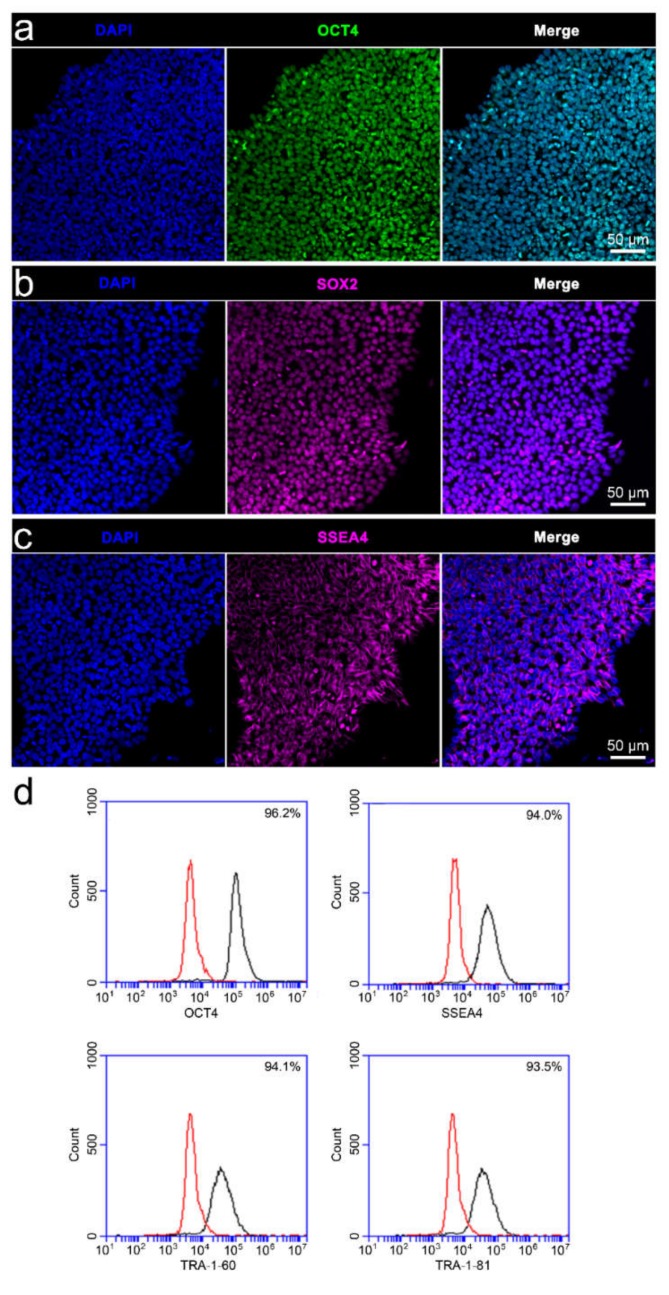
Immunophenotypic characterisation of the iPSCs: (**a**–**c**) iPSCs cultured on PPy:DBS substrate and stained with nuclear DAPI colocalised with pluripotency markers (**a**) Oct4, (**b**) SOX2, and (**c**) SSEA4. (**d**) Flow cytometry of the iPSCs confirming high level expresson OCT4, SSEA4, TRA-1-60, and TRA-1-8 (black histograms). Red histograms indicate the isotype controls. Scale bars as indicated.

**Figure 4:**
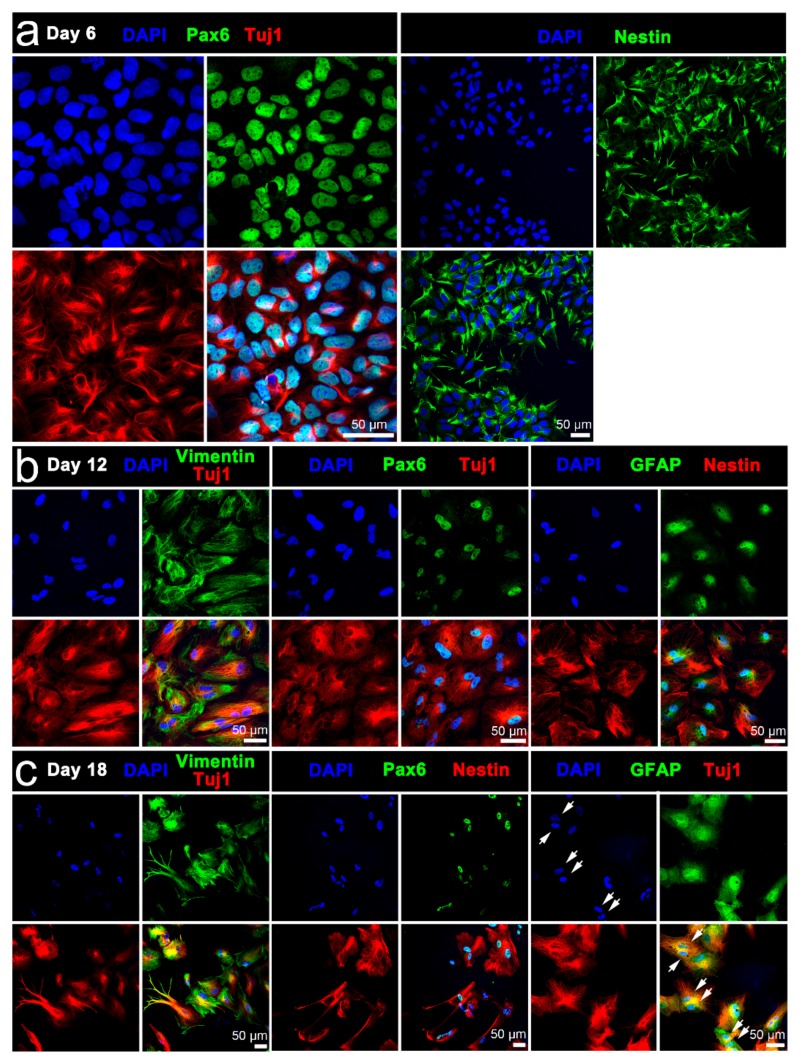
Immunophenotypic characterisation of iPSC differentiability: expression of neuroectodermal progenitor cell markers Pax6, nestin, and vimentin, early neuronal marker Tuj, and glial cell marker GFAP at (**a**) day 6, (**b**) day 12, or (**c**) day 18 of iPSC differentiation. Day 18 cell cultures were distinguished by more prominent neurite outgrowths emanating from Tuj1-expressing neurons. GFAP-expressing cells frequently appeared contigous with neurons, signified by two adjacent DAPI-labelled nuclei (arrows) and converged fluorescent labelling of adjoining glia and neurons. Scale bars as indicated.

Immunophenotyping confirmed the pluripotency of the iPSCs, with prototypical colonies on unstimulated PPy:DBS film consisting of high-density undifferentiated cells expressing transcriptional factors OCT4 and SOX2, and cell surface glycosphingolipid SSEA4 (Figure 3a–c). Moreover, flow cytometry of the iPSCs confirmed high level expression of OCT4, SSEA4, and cell surface antigens TRA-1-60 and TRA-1-81 (Figure 3d). The iPSCs readily differentiated to cells of neural lineage using the traditional neural induction method (Figure 4). More specifically, following 6 days of culture in neural induction medium, cultures consisted of large numbers of differentiating cells expressing neuroectodermal progenitor cell markers Pax6 and nestin, and early neuronal marker Tuj1 (Figure 4a), with increasing cell heterogeneity and maturation thereafter supported by GFAP-expressing cells indicative of glia, as well as polarized Tuj1 neurons exhibiting axons and dendritic arborizations (Figure 4b,c). Interestingly, GFAP-expressing cells frequently appeared contiguous with neurons, signified by two adjacent DAPI-labelled nuclei and converged fluorescent labelling of adjoining glia and neurons.

### 3.3. Characterisation of Stimulated iPSCs

#### 3.3.1. Conventional Electrical Stimulation with Chemical Induction

**Figure 5:**
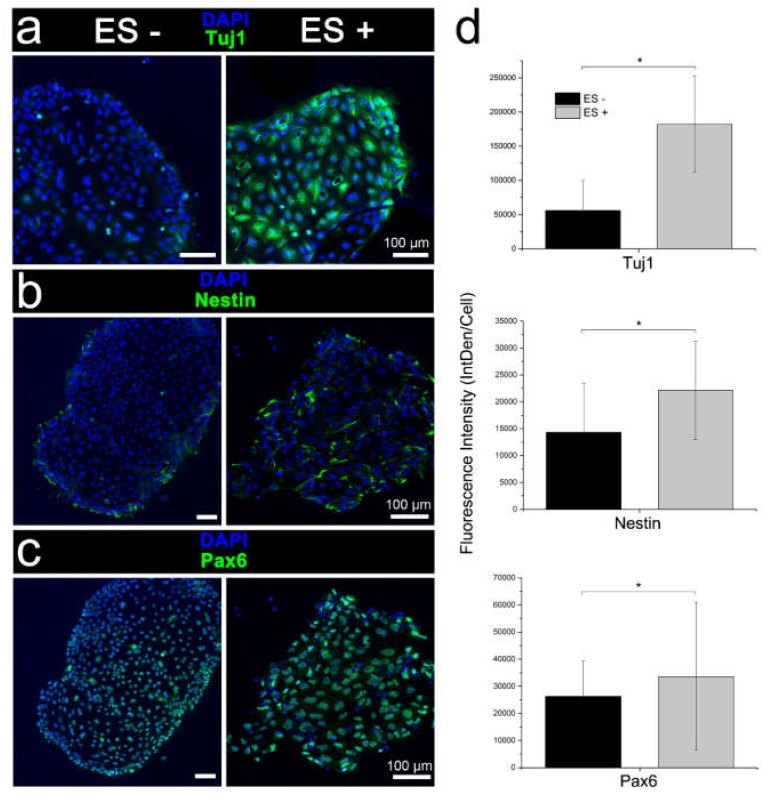
Immunophenotypic characterisation of iPSC differentiation following the conventional chemical induction with (ES+) and without (ES-) electrical stimulation. Expression of (**a**) early neuronal marker Tuj and neuroectodermal progenitor cell markers, (**b**) nestin, and (**c**) Pax6. Electrically stimulated cell cultures were distinguished by a higher antigen expression and diffuse nestin-labelled neurites outgrowths. Scale bars as indicated. (**d**) Quantitative assessment of Tuj1, nestin, and Pax6 immunocytochemistry (integrated density per cell; IntDen/Cell), confirming qualitative assessment. Mean ± S.D.; *n* = 3–5. One-way analysis of variance (ANOVA) with a Bonferroni post-hoc test. *p < 0.001.

Initial validatory studies on the conventional electrical stimulation of iPSCs affirmed differentiation in the neural induction medium with marked increases in expression of early neuronal cell marker Tuj1, and neural progenitor cell markers Pax6 and nestin compared to unstimulated cultures (Figure 5). 

#### 3.3.2. Electrical Stimulation without Chemical Induction 

**Figure 6:**
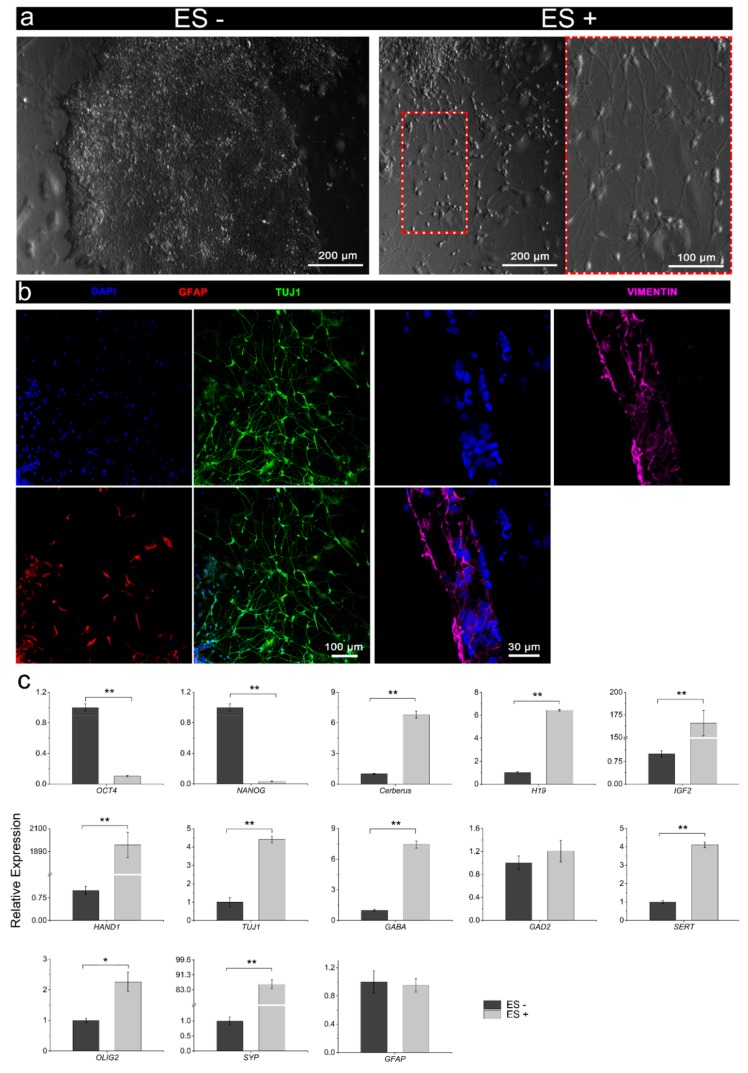
Characterisation of the iPSCs following electrical stimulation (ES+) versus no stimulation (ES-) without chemical inducers. (**a**) Bright-field micrographs of ES- (left panel) and ES+ (right panel) iPSC cultures, with ES- cultures consisting of classical iPSC-colonies with high-density undifferentiated cells, while ES+ cultures consisted of more dispersed and differentiated cells with clear neuronal morphology. ES+ cell cultures were distinguished by polarised neurons possessing dendritic arborizations and elongated axonal-like projections. (**b**) Immunophenotyping confirmed the neural induction of ES+ cultures, showing high-density Tuj1-expressing neurons with diffuse intersecting neurites, and to a lesser extent GFAP-expressing cells, as well as cells expressing the neuroectodermal progenitor cell marker vimentin. Scale bars as indicated. (**c**) Comparative gene expression (pluripotency: OCT4, NANOG; endodermal: Cerberus, H19; mesodermal: IGF2, HAND1; neuroectodermal: TUJ1, GABA, GAD2, SERT, OLIG2, SYP, GFAP) of the iPSCs following electrical stimulation (ES+) or without electrical stimulation (ES-). Relative gene expression represents data normalized to β-actin and expressed relative to the non-stimulated iPSCs. Mean ± S.D.; *n* = 3. One-way ANOVA with a Bonferroni post hoc test. *p < 0.01; **p < 0.001.

From a morphological assessment, there was a clear divergence between the unstimulated and stimulated iPSCs maintained in the stem cell culture medium (Figure 6a). The unstimulated cells continued to exhibit characteristic undifferentiated cell morphology, remaining as high-density populations of round cells with large nuclei, forming clearly defined colonies (Figure 6a, left panel). In contrast, electrically stimulated cultures principally consisted of polarised (mutlipolar and bipolar) cells with prominent outgrowths (Figure 6a, right panel). These outgrowths appeared to intersect, suggesting connections and networks. Immunocytochemistry of the stimulated cultures confirmed a neural cell phenotype, including Tuj1-expressing soma and neurites, and to a lesser extent, GFAP-expressing cells indicative of glia, as well as cells expressing the neuroectodermal progenitor cell marker vimentin (Figure 6b). RT-qPCR corroborated morphological assessment and immunophenotyping, whereby assessment of gene expression confirmed the downregulation of pluripotency markers OCT4 and NANOG for stimulated cells compared to the unstimulated iPSCs, and concomitant increased expression of endodermal (Cerberus, H19), mesodermal (IGF2, HAND1), and neuroectodermal (TUJ1, GABA, SERT, OLIG2, SYP, GFAP) markers (Figure 6c). Although not statistically significant, the higher expression of GAD2 following electrical stimulation was consistent with the increased expression of other neuroectodermal markers. Interestingly, the transcript for glial cell marker GFAP was low for both stimulated and un-stimulated cells (Figure 6c).

## 4. Discussion

Here, we describe the use of electrical stimulation for differentiating human iPSCs and expound the versatility of electroactive PPy:DBS. Initial CV and impedance studies critically demonstrated the conductivity and stability of PPy:DBS films necessary for iPSC stimulation, and affirm the method used for their preparation [5,13]. In addition, immunophenotyping of the iPSCs confirmed pluripotent cell state, with undifferentiated cells ubiquitously expressing pluripotency markers OCT4, SOX2, SSEA4, TRA-1-60, and TRA-1-81. Stemness of the iPSCs was also verified by their differentiability to neural cell lineage using the standard neural induction protocol. Notably, early to late expression of Tuj1 by differentiating cells is consistent with Tuj1 being present in mitotically active neuronal precursors, as well as newly formed immature postmitotic neurons and more differentiated neurons [16]. Finally, initial studies of conventional electrical stimulation of the iPSCs within neural induction medium affirms published reports of augmented cell differentiation using electrical stimulation contemporaneously with cell-specific induction media [5,6,7,8].

Having verified the performance of the platform for conventional stimulation, we investigated stimulation without chemical inducers. While RT-qPCR indicated stimulated iPSC cultures included cells of the three germ lineages—endoderm, ectoderm and mesoderm, transcriptional analysis together with morphological assessment and immunophenotyping indicated cells preferentially assumed a neuronal fate. Interestingly, relatively low glial cell induction was signified by immunophenotyping and RT-qPCR following electrical stimulation. The proclivity for neuronal rather than glial cell induction is consistent with previous reports showing stimulation biases native human NSCs and murine ESCs towards neuronal lineages [5,6,7]. This finding is also consistent with in vitro and in vivo effects of direct current electrical stimulation on neural plasticity being primarily attributed to the direct modulation of neurons [17]. However, although glial cells are unable to generate action potentials, they are electrically active, undergo membrane potential changes in response to neuronal activity [18,19], and are potentially sensitive to voltage changes that may include electrical stimulation [17]. Notwithstanding the potential for direct modulation of glial function by external electrical stimulation, an effect on the antecedent stem cell induction to glia remains undetermined, although further investigation is warranted.

Evidently, neural commitment and ensuing differentiation is a complex process, with the mechanisms governing differentiation still relatively poorly understood. Notably, progress is being abetted by the advent of pluripotent stem cell-based modelling, beginning with human embryonic stem cells and more recently using iPSCs [9,10,11,12]. Remarkably, human iPSCs are readily obtainable and self-renewing cells, overcoming previous limitations of, for example, non-human animal modelling, not least because of limited availability and the heterogeneity of cells restricting critical temporal analysis of neurogenesis.

The ability to model human neural development using iPSCs does, however, require effective methods of directing stem cell differentiation to neural lineage. Protocols ideally need to recapitulate temporal differentiation through different stages of neural development, including regulation mechanisms and signaling pathways of early cell fate specification and later transition to the myriad of end-stage neuronal and glial cell types. The design of optimized neural induction protocols has largely entailed the development of specialized culture media, enabling generation of neurons and, in some instances, supporting neuroglia as occurs in vivo, with the latter circumventing the need to introduce cells through co-culture [20,21,22].

Our simplified differentiation protocol yields iPSC-derived neuronal, and to a lesser extent, glial cells without the need for separate cell co-culture or specialized media. The high to lower ratio of neurons and glia mirrors our earlier published findings for both 2D PPy:DBS mediated and 3D PEDOT:PSS mediated electrical stimulation of human NSC differentiation [5,6]. Importantly, while we anticipate the present protocol to be of broad applicability for both research and translation, future research directions could include modification of electrical stimulation regimen to modulate cell fate specification, including non-neural cell induction. In addition, investigation into the fundamental molecular mechanisms underlying the effects of stimulation that governs differentiation will be useful. For example, brain-derived neurotrophic factor (BDNF), a member of the neurotrophin family of growth factors and important for neuronal maturation, growth, and survival, has been shown to be responsive to electrical stimulation [23]. Correspondingly, electrical stimulation elevates cell-surface expression of BDNF-receptors (tyrosine receptor kinase B; TrkB) of central nervous system neurons in vitro [24,25], and enhances the trophic effects of BDNF on neurons in vivo [26]. Although the involvement of BDNF in stimulating iPSCs remains to be determined, animal cell modeling suggests increased BDNF expression by first initiating an action potential that triggers calcium ion influx via voltage-gated calcium channels and subsequent activation of Ca²⁺/calmodulin-dependent protein kinase II (CaMKII), ensuing phosphorylation of cyclic adenosine monophosphate (cAMP)-response element binding protein (CREB), culminating in transcription of the BDNF gene [27,28]. Recruitment of intracellular TrkBs to the plasma membrane of cells is believed to be “gated” by the key intracellular signal transducer cAMP following stimulation [25].

Acceding the opportunities for further investigation, we propose the present platform to be of considerable utility, being amenable for advanced research and translation, with the former including identifying novel mechanisms of fate regulation, as well as advanced tissue engineering in vitro, innovating electroceuticals, and regenerative medicine.

## Figures and Tables

**Table 1:** Primers used for RT-qPCR.

Name	Forward	Reverse	Length (bp)	
*OCT4*	CAATTTGCCAAGCTCCTGA	CGTTTGGCTGAATACCTTCC	105	
*NANOG*	TACCTCAGCCTCCAGCAGAT	TGCGTCACACCATTGCTATT	146	
*Cerberus*	GCCATGAAGTACATTGGGAGA	CACAGCCTTCGTGGGTTATAG	69	
*H19*	GCAAGAAGCGGGTCTGTTT	GCTGGGTAGCACCATTTCTT	105	
*HAND1*	AAGCGGAAAAGGGAGCTG	ACTCCAGCGCCCAGACTT	112	
*IGF2*	CTGTTTCCGCAGCTGTGAC	GGGGTATCTGGGGAAGTTGT	118	
*SERT*	CTCCGAGGACAACATCAC	CTTGCCAGAGGTCTTGAC	199	
*GABA*	GTCCAGGTCTGTCTGACTGTCTT	CTTCACTTCGGTTACACGCTCTC	197	
*SYP*	TTGCCTTCCTCTACTCCAT	GCCATCTTCACATCTGACA	172	
*TUJ1*	ACACAGGCGTCCACAGTT	GTTCCAGGTCCACCAGAATG	167	
*OLIG2*	TTGCTCCTCTTCCTCCTT	GGCTTCCAACTAACTTGTG	129	
*GFAP*	ATCAACTCACCGCCAACA	CTTCATCTGCTTCCTGTCTATA	153	
*GAD2*	CGACCTGCTCCAGTCTCCAA	ATGCCGCCCGTGAACTTCT	144	
*β-Actin*	AGGCATCCTCACCCTGAAGTA	CACACGCAGCTCATTGTAGA	103	
